# Giant cell glioblastoma in 6‐year‐old kid: Report of an unusual case

**DOI:** 10.1002/ccr3.3102

**Published:** 2020-09-14

**Authors:** Suraj Shrestha, Sushan Homagain, Akash Raut, Gopal Sedhain, Suraj Bhatta, Shreya Shrivastav

**Affiliations:** ^1^ Maharajgunj Medical Campus Institute of Medicine Kathmandu Nepal; ^2^ Department of Neurosurgery Tribhuwan University Teaching Hospital Kathmandu Nepal; ^3^ Department of Pathology Tribhuwan University Teaching Hospital Kathmandu Nepal

**Keywords:** giant cell glioblastoma, glioblastoma multiforme, pediatrics

## Abstract

Pediatric giant cell glioblastoma, a highly malignant and lethal tumor, can only be distinguished from glioblastoma multiforme histologically. Though it is said to have a better prognosis, adequate evidence in favor is lacking. Early diagnosis with gross total resection and adjuvant chemotherapy might increase the survival period.

## Graphical abstract

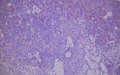

## 1 INTRODUCTION

Pediatric brain tumors, the most common solid tumors in children, stand second among all childhood malignancies. Supratentorial tumors are more common in early ages of life and early adolescence, whereas infra‐tentorial tumors such as ependymoma and medulloblastoma are more common in between these age groups.^1^

Glioblastoma multiforme (GBM) is the most common glial tumor of the brain associated with high mortality and morbidity; nevertheless, the giant cell glioblastoma (GCG) subtype, an unusual variant of glioblastoma is distinct in features on histopathology and supposedly has a better prognosis.

Recent studies report for an incidence of 1% in adults and 3% in children among all glioblastoma cases. We report a case of GCG in a 6‐year‐old boy along with a review of relevant literature.

## 2 CASE REPORT

**FIGURE 1:**
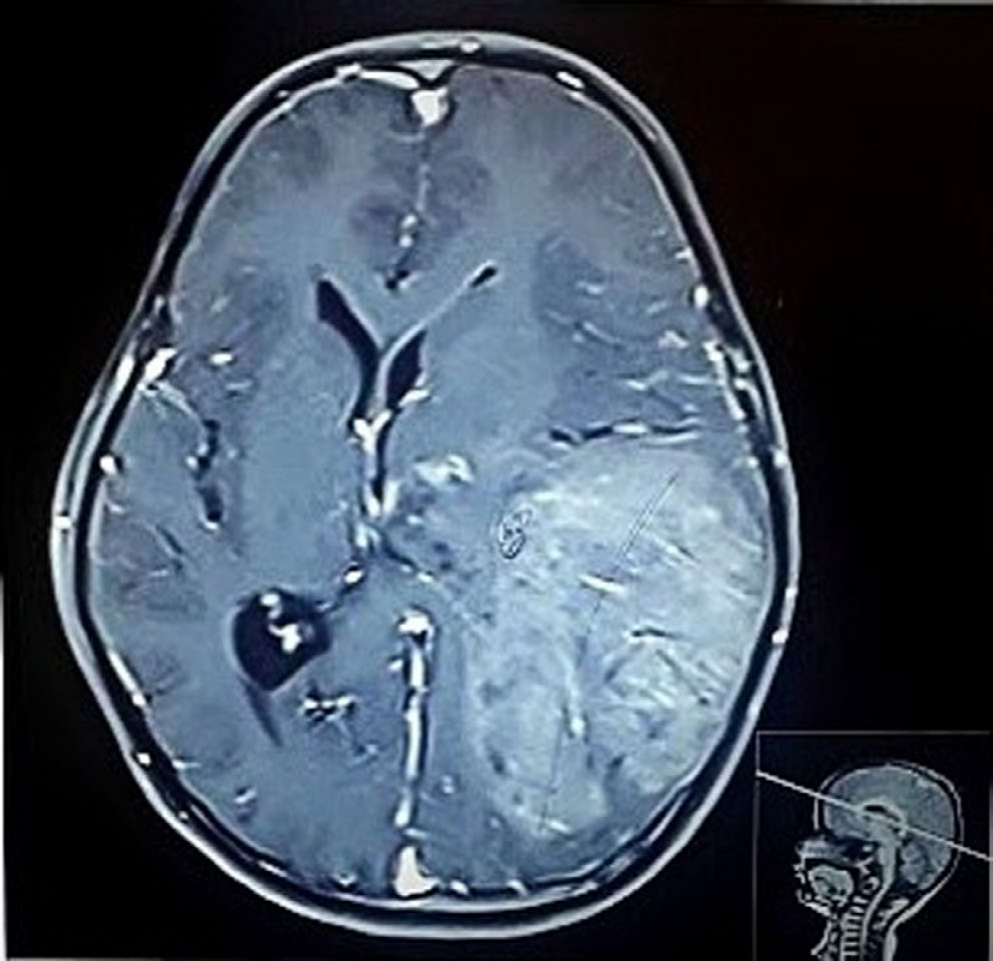
Contrast MRI brain showing a large heterogeneous enhancing lesion in left parieto‐occipital region reaching up to ventricle

**FIGURE 2:**
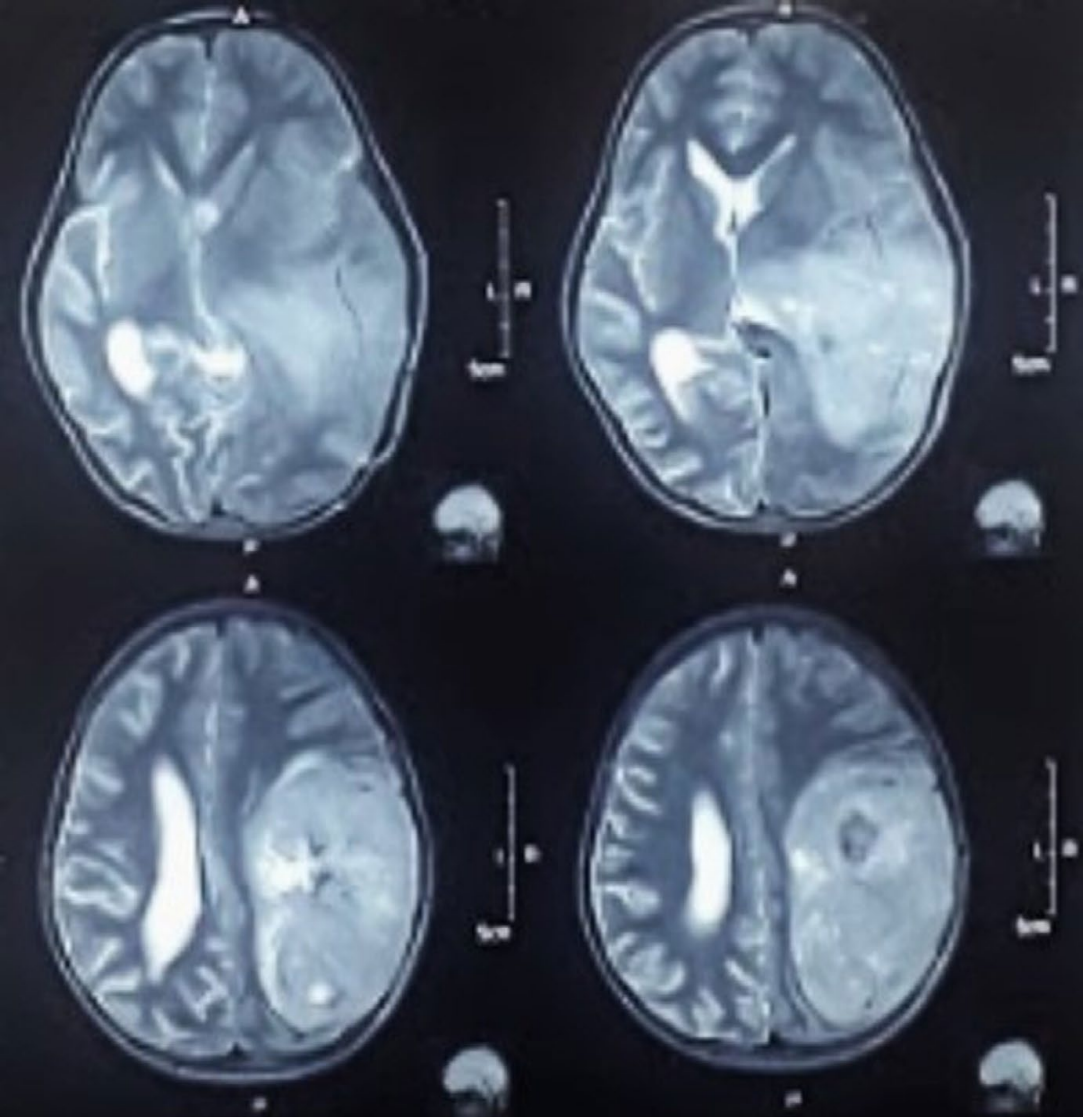
T2 axial images showing large hyperintense lesions

**FIGURE 3:**
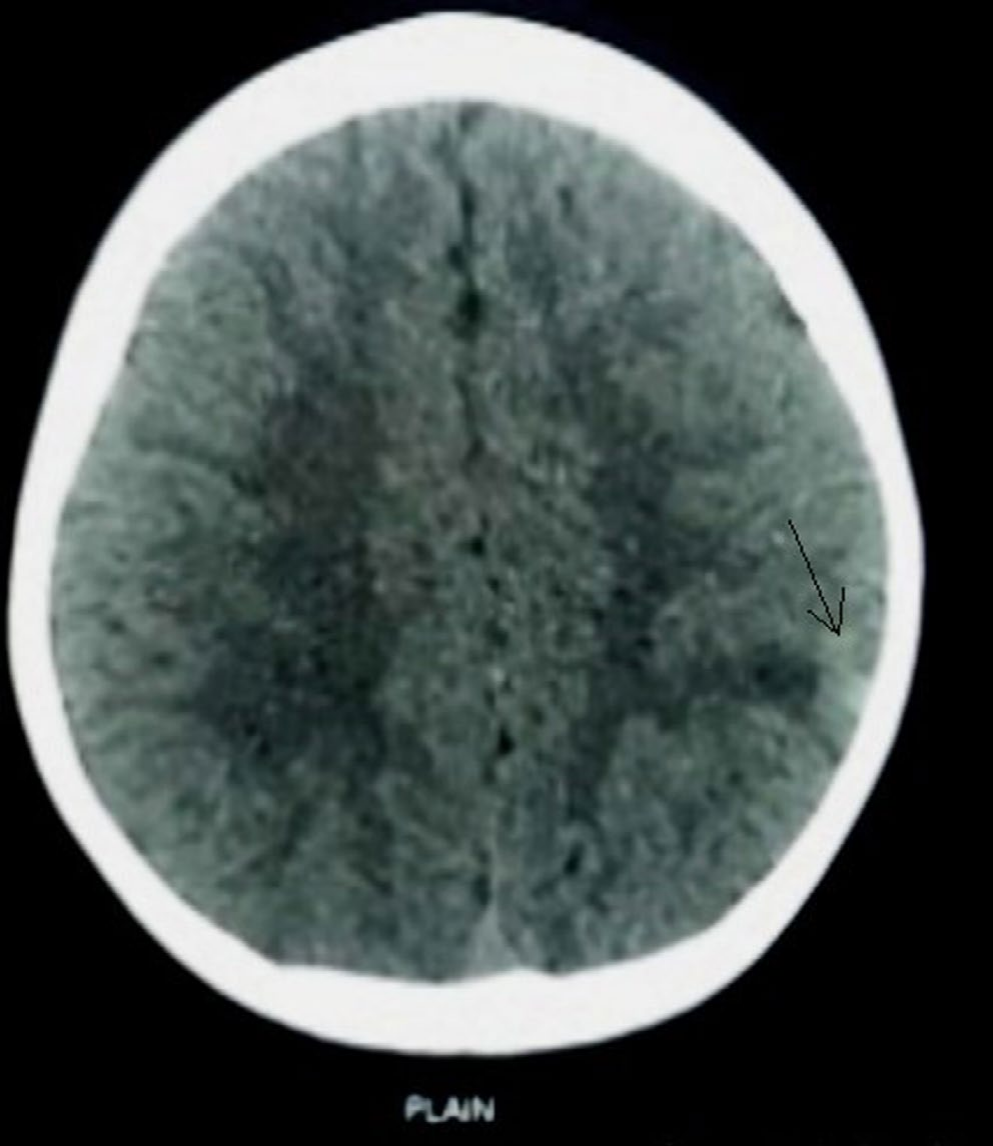
NCCT head revealing hyperdensity with small surrounding hypodensity in left high parietal region (arrow)

A 6‐year‐old young school‐going boy presented to the emergency department with a history of holocranial and progressively worsening headache, multiple episodes of projectile nonbilious vomiting, and right‐sided weakness for 2 days. On neurological examination, there was a right sided hemiparesis with a power of at least ⅗ (Medical Research Council Grading) on flexors and extensors of shoulder, elbow, hip, knee, and ankle. Radiological investigation (magnetic resonance imaging) revealed a large heterogeneous contrast‐enhancing lesion in the left parietal region reaching up to the temporal horn medially and dura mater laterally (Figures 1 and 2), radiological features which were otherwise inapparent in the previous scans taken 9 months ago for generalized tonic clonic seizure. Plain computed tomographical (CT) scan done during that presentation showed a subtle ill‐defined hyperdense lesion on the left high parietal region. (Figure 3) On this basis, the diagnosis of viral encephalitis was made at another center and he was treated with acyclovir and was started on levetiracetam.

**FIGURE 4:**
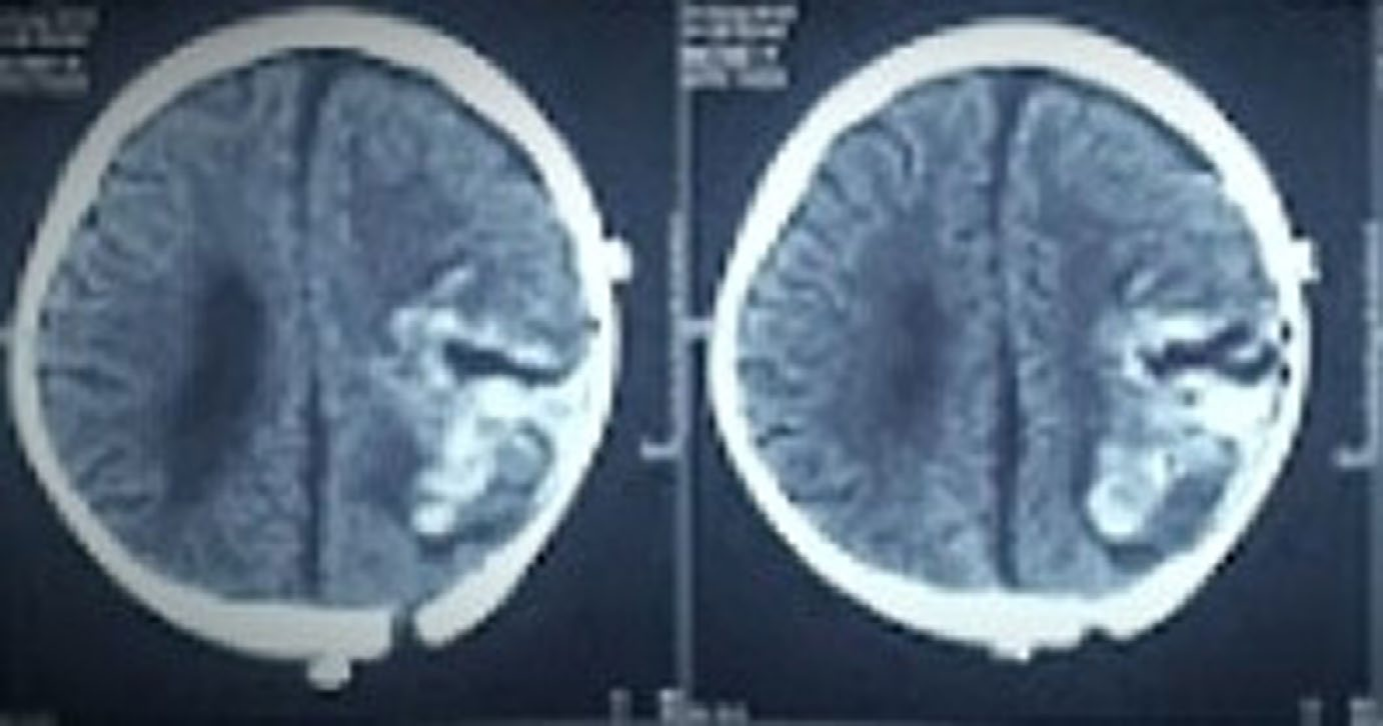
Post‐op CT scan showing hematoma at the operative site

In view of the short history and aggressive nature on radiology, a preoperative diagnosis of malignant brain pathology was made and surgery was planned. A Mitre's flap and a parieto‐occipital craniotomy were performed. There was a large yellowish soft vascular and suckable tumor with well‐defined margins adherent to the inner layer of the dura. As the tumor was vascular, and the patient became hemodynamically unstable, only subtotal excision of the tumor could be achieved. (Figure 4).

Postoperatively, he was ventilated for a day and woke up with no added deficits. The patient was discharged on the 8th postoperative day. The patient did not receive any form of radiotherapy/chemotherapy because his parents were reluctant as the disease was malignant and had a poor prognosis. The patient was kept on follow‐up in the Outpatient Department. The patient succumbed to the illness 4 months after surgery.

**FIGURE 5:**
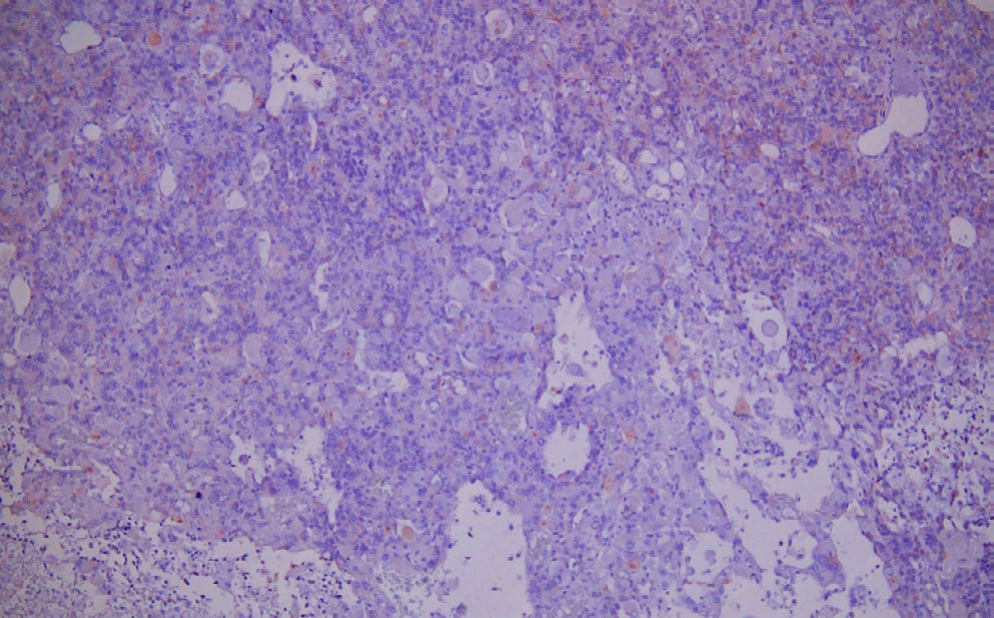
Histopathology of the excised mass showing multinucleated tumor giant cells with areas of necrosis (40× magnification)

**FIGURE 6:**
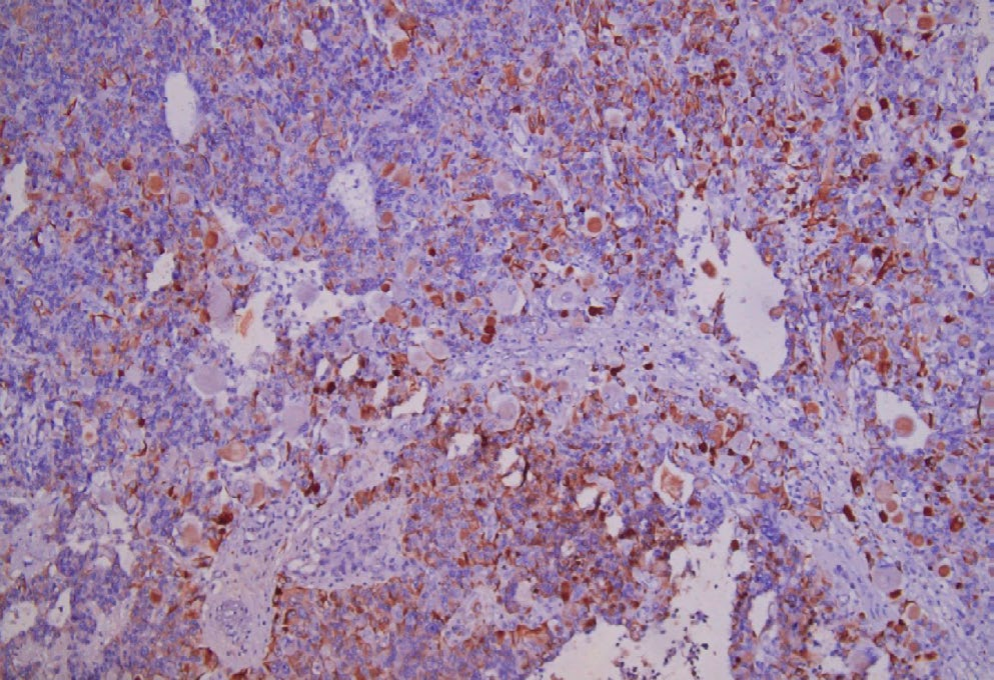
Immunohistochemistry of tumor: Positive for GFAP and Vimentin, negative for EMA. (40× magnification)

Histopathology of the tumor mass revealed diffusely infiltrating tumor with large tumor cells along with large nuclei and coarse chromatin. Many interspersed multinucleated tumor giant cells with bizarre nuclei, irregular dense chromatin, and prominent nucleoli are also seen. Large areas of palisading and necrosis and vascular proliferation are also noted with frequent mitosis along with atypia. (Figure 5) Immunohistochemistry of the tumor cells revealed glial fibrillary acidic protein (GFAP) positive, EMA (epithelial membrane antigen) negative, and vimentin positive. All these findings were suggestive of giant cell glioblastoma (WHO Grade IV). (Figure 6).

## 3 DISCUSSION

Giant cell glioblastoma (GCG), previously called monstrocellular brain tumor, a subtype of GBM is an uncommon neoplasm characterized by a predominance of bizarre multinucleated giant cells with abundant eosinophilic cytoplasm. WHO has classified GCG as a Grade IV tumor. However, being prognostically better in terms of survival than classical Grade IV GBM, it is not inappropriate to consider it as midway between grade III and grade IV gliomas.^2^ The definitive diagnosis of this tumor is based on its histological findings and patterns. Unfortunately, due to its rarity, the epidemiology, natural history, follow‐up, immunohistochemical, and cytogenetical analysis of this entity remains to be elucidated.^3^

Pediatric giant cell glioblastoma is considered extremely rare.^2^ In the literature, there are only around 100 reported cases of Pediatric GCG. GBM constitutes only about 5%‐10% of all the intracranial neoplasms in the pediatric age group and GCG’s are 3%‐5% of GBM’s at this age group and about 0.8% of all brain tumors.^2^ A study has shown the median age to be 11 years for pediatric GCG, but it affects the patients of wide age group ranging from children to young adolescents (4‐17 years).^4^ Often occurring in younger patients with variably reported male predominance, GCG’s are frequently supratentorial and more so in the temporal lobe, although there is no specific localization of the tumor. The frontal lobe, lateral ventricles, parietal lobe, optic chiasm, and cerebellum are less common sites, and the tumor is rarely multifocal.^5^

The classic “glioblastoma” and GCG have similar clinical presentations. Signs of raised intracranial pressure are often principal symptoms of GCG in the majority of pediatric patients presenting with headache and vomiting, as in our case. Hemiparesis is another common presenting symptom, being prevalent in 50% of patients. Seizures can be another early symptom in a minority of patients.^6^ The boy had seizure episodes 9 months back, diagnosed as viral encephalitis, and treated accordingly. This episode of seizure can be considered as an initial symptom of the tumor, but as there were no definitive radiographic findings suggestive of an intracranial space‐occupying lesion, it could be a mere coincidence. Due to these nonspecific symptoms, the tumor can mimic infections, inflammatory processes, and circulatory and immunological diseases.^7^ However, the duration of symptoms is often short.^6^ Pleomorphic xanthoastrocytoma (PXA) is an important clinical and histological differential diagnosis. Clinically, quicker evolution of seizures and histologically, numerous giant cells with multiple and atypical mitoses favors GCG. Immunohistochemical profiles such as neuronal antigens and p53 can help differentiate these two entities with GCG positive for p53 and negative for neuronal nuclear antigen, neurofilament protein, and synaptophysin.^8^ In some cases, GCG is associated with disorders of genetic origin‐like neurofibromatosis type 1 and tuberous sclerosis.^9^ Accordingly, the other differential diagnosis considered was subependymal giant cell astrocytoma. But it was unlikely as features of tuberous sclerosis were absent, and histologic features were rather suggestive of a high‐grade tumor. MRI of the brain usually reveals a contrast‐enhancing heterogeneous mass, with solid and cystic areas, hypointense on T1, and hyperintense on T2 sequences with surrounding edema.^2^ Intraoperatively, the tumor has been described as friable, moderately vascularized, amenable to suction, partially cystic and with a good cleavage plane.^10^ Like our case, dural adhesion of tumor can be seen in some.^11^

As mentioned earlier, GCG is diagnosed based on histopathological examination of the tumor mass. Microscopically, they are highly cellular lesions with abundant bizarre giant cells with nuclei of varying sizes, shapes, and numbers with areas of necrosis, mainly in a pseudopalisading or large ischemic forms. The tumor cells are positive for glial fibrillary acidic protein (GFAP) which represents the glial origin of the tumor. Immunohistochemistry studies have also shown positivity for S‐100, vimentin, alpha‐1 antichymotrypsin.^12^

There is no definitive surgical management protocol for this tumor due to its rarity. However, maximum safe resection along with adjuvant radiotherapy can improve survival rate from 5 to 13 months, similar to GBM patients.^13^ Some studies suggest the increased survival rate among GCG patients compared to GBM due to younger presenting age. The more visible and circumscribed margins of GCG leading to better resection could be another contributing factor.^13^, ^14^ However, a study of 18 pediatric patients by Karremann et al. showed no significant difference in median age, male preference, median clinical history, and prognosis between GCG and GBM.^4^ Patients who did not have a gross total resection (GTR) have a higher mortality rate.^15^ Deep and infiltrating tumors with difficult resectability had a bad prognosis even with chemotherapy and radiotherapy. Therefore, the more superficial and localized the tumor, the better the prognosis.^16^ On these accounts, subtotal resection of tumor without adjuvant chemotherapy/radiotherapy might have led to the early demise of the patient in our case. However, Taemin and Rutkowski found overall mortality of 75% and a median time to death of 13.1 months for gross total resection, and a mortality rate of 93% and a median time to death of 15.4 months in the subtotal resection, which could not reach statistical significance.^17^

## 4 CONCLUSION

Giant cell glioblastoma is an uncommon subtype of glioblastoma multiforme which is even uncommon in the pediatric population. Differentiating it from GBM on a clinical basis is difficult because of similar characteristics and is entirely based on histopathological examination. There is no established consensus that GCG has a better prognosis as literature with varying conclusions are published. The case presented shows how a subtle ill‐defined lesion mimicking infection progressed rapidly to a huge fatal tumor over a short time making the complete removal unfavorable. Early diagnosis with total resection of the tumor and adjuvant chemotherapy may increase the survival of the patient.

## CONFLICT OF INTEREST

None.

## AUTHORS CONTRIBUTIONS

Suraj Shrestha, Sushan Homagain, Akash Raut, Suraj Bhatta: were involved in writing of the manuscript. DrGopal Sedain and Dr Shreya Shrivastav: reviewed the manuscript. All the authors were involved in final reviewing of the manuscript.

## INFORMED CONSENT

Written informed consent was taken from the patient's parents before writing the manuscript.
